# MiR-1290 promotes myoblast differentiation and protects against myotube atrophy via Akt/p70/FoxO3 pathway regulation

**DOI:** 10.1186/s13395-021-00262-9

**Published:** 2021-03-15

**Authors:** Ji Che, Cuidi Xu, Yuanyuan Wu, Peiyu Jia, Qi Han, Yantao Ma, Xiaolei Wang, Yongjun Zheng

**Affiliations:** 1 Department of Pain, Huadong Hospital, Shanghai Key Laboratory of Clinical Geriatric Medicine, Fudan University, No. 221, West YanAn Rd, Shanghai, 200040 P.R. China; 2 Department of Osteoporosis and Bone Disease, Huadong Hospital, Research Section of Geriatric Metabolic Bone Disease, Shanghai Geriatric Institute, Shanghai, China

**Keywords:** miR-1290, Myoblast differentiation, Atrophy, Akt/P70/FoxO3

## Abstract

**Background:**

Sarcopenia is a common skeletal disease related to myogenic disorders and muscle atrophy. Current clinical management has limited effectiveness. We sought to investigate the role of miR-1290 in myoblast differentiation and muscle atrophy.

**Methods:**

By transfecting miR-1290 into C2C12 cells, we investigated whether miR-1290 regulates myogenesis and myotube atrophy via AKT/P70 signaling pathway. MHC staining was performed to assess myoblast differentiation. Differentiation-related MHC, Myod, and Myog protein levels, and atrophy-related MuRF1 and atrogin-1 were explored by western blot. An LPS-induced muscle atrophy rat model was developed. RT-PCR was conducted to analyze miR-1290 serum levels in muscle atrophy patients and normal controls (NCs).

**Results:**

The miR-1290 transfection increased MHC-positive cells and MHC, Myod, and Myog protein levels in the miR-1290 transfection group, demonstrating that miR-1290 promoted C2C12 myoblast differentiation. Myotube diameter in the miR-1290 transfection group was higher than in the TNF-α-induced model group. Western blot analysis showed decreased MuRF1 and atrogin-1 levels in the miR-1290 transfection group compared with the model group, demonstrating that miR-1290 protected against myoblast cellular atrophy. Luciferase assay and western blot analysis showed that miR-1290 regulation was likely caused by AKT/p70/FOXO3 phosphorylation activation. In the LPS-induced muscle atrophy rat model, miR-1290 mimics ameliorated gastrocnemius muscle loss and increased muscle fiber cross-sectional area. Clinically, miR-1290 serum level was significantly decreased in muscle atrophy patients.

**Conclusions:**

We found that miR-1290 enhances myoblast differentiation and inhibits myotube atrophy through Akt/p70/FoxO3 signaling in vitro and in vivo. In addition, miR-1290 may be a potential therapeutic target for sarcopenia treatment.

## Background

Skeletal muscle is a crucial component of the human body, occupying about 40–50% of body mass [1]. Skeletal muscle has exercise, metabolism, and endocrine functions [2] and participates in most activities of the human body. Abnormal skeletal muscle causes a marked decrease in quality of life and presents a great medical burden on the population, especially elders [3]. Muscle atrophy is a common skeletal disease characterized by muscle wasting or loss of muscle fibers. The causes of muscle atrophy include metabolic diseases or toxins, such as diabetes, AIDS, toxic drugs, and insufficient kidney function [4]. To date, clinical strategies to treat skeletal muscle atrophy have included surgery, such as free muscle transfer; as well as pharmacology intervention; rehabilitation, such as radiation, muscle stimulation, and manual therapy; and myogenic stem cell transplantation. However, despite numerous advances in the past, current clinical strategies have not achieved full muscle function restoration [5]. Furthermore, the above treatments are often accompanied by side effects [6].

Commonly, muscle atrophy can be divided into decreased muscle protein synthesis and increased proteolysis [6]. The decreased regenerative capacity of the muscle plays crucial roles in the complex process of muscle atrophy. Myogenesis initializes the proliferation and differentiation of myoblasts into muscle cells, and consequently the fusion of muscle cells into myotubes, which in turn leads to the maturation of muscle fibers [7]. miRNAs are noncoding single-stranded RNAs that can block translation or elicit degradation of target genes [8]. At present, targeting miRNAs is regarded as a new therapeutic approach for muscle activities, such as MiR-133 [9], miR-222 [10], and MiR-204-5p [11]. A special miRNA, miR-1290, has been reported to be associated with nasopharyngeal development [12], lung cancer biomarkers [13], and pulmonary fibrosis [14]. However, its role in the regulation of C2C12 myogenic differentiation and myotube atrophy, as well as potential-related mechanisms, has not been elucidated.

In this study, we demonstrated that miR-1290 enhanced C2C12 myogenic differentiation and inhibited myotube atrophy in vitro by targeting FOXO3 via the AKT/P70 pathway. Furthermore, miR-1290 ameliorated the loss of gastrocnemius muscle and increased muscle cell area in an LPS-induced muscle atrophy rat model. Additionally, miR-1290 was decreased in muscle atrophy patients.

## Materials and methods

### Materials

Phosphate-buffered saline (PBS), Dulbecco’s Modified Eagle’s Medium (DMEM), penicillin-streptomycin, and fetal bovine serum were provided by Thermo Fisher (Thermo Fisher Scientific, CA, USA). TNF-α (AF-315-01A) was obtained from PeproTech. Protein extraction Kit, BCA Protein Assay kit, Alexa Fluor 488-labeled goat anti-mouse IgG, and Nuclear and Cytoplasmic Protein Extraction kit were purchased from Beyotime Institute of Biotechnology (Beyotime, Haimeng, China). Trizol was purchased from Sigma-Aldrich (St. Louis, MO, USA). Lipofectamine 2000 (Invitrogen, Waltham, MA, USA) was purchased from Invitrogen. The miR-1290 mimic and negative control were synthesized by GenePharma (Shanghai, China). Akt inhibitor GDC-0068 (#HY-15186) was purchased from MedChemExpress (Suite Q, NJ, USA). Primary antibodies against myosin heavy chain (MHC) (sc-376157, 1:500), Akt (sc-5298; 1:500), P-P70 (sc-8416; 1:500), and P70(sc-8418; 1:500) were purchased from Santa Cruz Biotechnology (Dallas, TX, USA). FOXO3A (10849-1-AP, 1:1000), MyoG (67082, 1:1000), MyoD (18943-1-AP, 1:1000), Lamin B1(12987-1-AP, 1:5000), and GAPDH (60004-1-Ig, 1:5000) were obtained from Proteintech (Wuhan, China). MuRF1 (ab183094; 1:5000) and atrogin-1 (ab168372; 1:5000) were purchased from Abcam (Cambridge, MA, USA). Antibodies specific for p-AKT were obtained from Cell Signaling Technology (Danvers, MA, USA). Secondary antibodies were provided by Proteintech (Wuhan, China). All other chemicals not mentioned were of analytic grade.

### Cell culture

The mouse myoblast C2C12 cells were provided by Cyagen Biosciences, Inc., and cultured in DMEM containing 1% penicillin-streptomycin and 10% fetal bovine serum following the American Type Culture collection instructions. Cells were maintained in an incubator at 37 °C with 5% CO_2_ for regular maintenance. For the differentiation stage, we changed pre-confluent C2C12 cultures from high-serum (10% FBS) to low-serum conditions (2% HS) to induce cell cycle exit, commitment to myogenic differentiation and fusion between myoblasts to form multinucleated myotubes. Normally, during C2C12 differentiation, myotube density increased sharply 5 days after confluence. For the differentiation study, C2C12 cells with miR-1290 mimic were transfected with Lipofectamine 2000 and set as the transfection group, while the C2C12 cells were set as the control group. For the C2C12 myotube atrophy study, miR-1290 mimic was transfected into C2C12 cells by Lipofectamine 2000 following the manufacturer’s instructions. After 24 h, TNF-α (20 ng/ml) was added. The C2C12 cells were cultured for 24 h. After transfection of miR-1290 mimic, cell differentiation was induced by substituting the regular maintenance medium with the differentiation medium (DMEM + 2% horse serum).

### C2C12 cell differentiation assay

The MHC immunofluorescence (IF) assay was employed to investigate the differentiation role of miR-1290 in C2C12 cells [15]. C2C12 cells were cultured in six-well plates with prepared coverslips [16]. After adding miR-1290 mimic, the C2C12 cells on coverslips were carefully washed using PBS two times, then incubated in 0.25% Triton X-100 for 15 min. Subsequently, the C2C12 cells were blocked using blocking reagent, incubated with primary anti-MHC overnight, then incubated in Alexa Fluor 488-labeled goat anti-mouse IgG for 2 h. Finally, the cells were counterstained by DAPI for 10 min. The images were processed using Image Pro Plus 6.0. The ratio of MHC-positive myotube area versus the total area was observed.

### Giemsa staining and measurement of myotube diameters

C2C12 myotube diameter was measured as previously reported. Cells were first washed with PBS twice and fixed in 4% paraformaldehyde for 10 min, after which the myotubes were stained with 10% Giemsa solution for 40 min and then observed by an optical microscope (Leica). For each condition, six pictures were randomly taken from each well of the six-well plates. The diameters of three different sites in each myotube were measured using ImageJ software, and at least 100 myotubes in one well were measured.

### Dual-luciferase reporter assay

To verify whether FOXO3 was the direct target gene of miR-1290, luciferase analysis was performed. FOXO3 3’UTR psiCHECK-2 vector containing miR-1290 predicted seed match site or mutant site were purchased from HANBIO (Shanghai, China). HEK293T cells were cultured in 12-well plates and co-transfected with luciferase reporter vector and miR-1290 using the Lipofectamine 2000 following the guidance of a previous report [17]. After 48 h of co-culture, the C2C12 cells were harvested and washed twice using PBS. The dual-luciferase assays were employed to explore the fluorescence intensity. Luciferase activity was assessed via a Dual-Luciferase Reporter Assay System (Promega), and the data were normalized to the control, which was the β-gal luciferase activity levels, before data calculation.

### Qualitative real-time polymerase chain reaction (RT-PCR)

RNA was isolated by Trizol (Takara) and obtained using a miRNeasy Mini kit (QIAGEN) in accordance with the manufacture manual. Reverse transcription was performed using a TaqMan microRNA cDNA Synthesis kit (MT006) in accordance with the manufacturer’s instructions. Each qPCR reaction solution was prepared using 50 ng of cDNA, TB Green® qRT-PCR (1×) in a total volume of 20 μl with 40 cycles of 5 s at 95 °C, 20 s at 60 °C, and 10 s at 95 °C by Applied Biosystems® StepOne RT-PCR system. The housekeeping gene U6 was run and used as an internal standard for RT-PCR assay with primer: (forward 5′-GGAACGATACAGAGAAGATTAGC-3′, reverse 5′-TGGAACGCTTCACGAATTTGC-G-3′).

### Western blot

Western blotting was performed to explore the related protein activities in the C2C12 cells. In brief, C2C12 cells after exposure were harvested, and protein of the cells was isolated. The protein concentration was estimated by BCA kits and adjusted. Subsequently, protein was separated and transferred to PVDF membrane. The PVDF membrane was blocked using blocking reagent and incubated with primary antibodies separately overnight at 4 °C. Then, the PVDF membrane was subjected to incubation with desired secondary anti-rabbit antibody. GAPDH was used as the internal control. The intensity was analyzed by ChemDoc.

### RNA interference

The siRNA was transfected into C2C12 cells by Lipofectamine 2000 following the manufacturer’s instructions. After transfection, the cells were cultured with growth medium or induction medium for differentiation for 4 days. si-FoxO3-1: TGGACGACCTGCTGGATAA, si-FoxO3-2: CCGAGAACCTCATGGACGA.

### Animal experimental design

All animal experiments were approved and followed by the Institutional Animal Care and Use Committee of Fudan University. Sprague Dawley (SD) rats (150–160 g, 6 weeks old, specific pathogen-free (SPF), healthy, and all males) were obtained from Shanghai Sippr BK Laboratory Animals, Ltd., and housed in Fudan University at a temperature of 20–25 °C, humidity of 40% ± 5%, and in a 12-h light/12-h dark cycle environment at SPF grade. Rats were provided with free access to adequate pellets, and diet and water were SPF grade. A total of 20 rats were distributed in two different groups. All rats were injected with LPS to establish the atrophy model group, and 10 were set as the control group and intramuscularly injected with miR-NC, while 10 were set as the treatment group with miR-1290 mimic. LPS (50 μg/kg) dissolved in saline was injected intraperitoneally into the rat to induce atrophy following previously described methods [18]. The miR-NC and mir-1290 mimic were administrated 7 days after model establishment. After last administration, rats in all groups were euthanized after anesthesia, the gastrocnemius muscles were harvested, and the gastrocnemius muscle weight (GW) and gastrocnemius muscle weight/body weight (GW/BW) ratio were recorded. Then, the gastrocnemius was fixed using 10% paraformaldehyde, embedded, and cut into slices at 5 μm. The slices were stained using hematoxylin and eosin (HE), then imaged using a microscope (MF31, Mshot, Guangzhou, China). The myofiber cross-sectional area was determined following a previous report.

### Clinical serum sample preparation

Serum samples were obtained from 12 knee osteoarthritis patients with muscle atrophy and 6 patients without muscle atrophy as normal controls. For all patients, lower limb muscle strength was measured by a manual dynamometer, and lean mass was also measured.

### Analysis of data

Cell experiments were repeated three times. The results were shown as mean ± standard deviation (SD) and determined using one-way analysis of variance (ANOVA) to analyze statistical significance. *P* value < 0.05 was considered statistically significant. All statistical analyses were performed using statistical software (SPSS22.0, Chicago, IL, USA) and are shown by GraphPad Prism 6.0 (GraphPad Software Inc., San Diego, CA, USA).

## Results

### MiR-1290 expression is negatively correlated with muscle atrophy

**Fig. 1:**
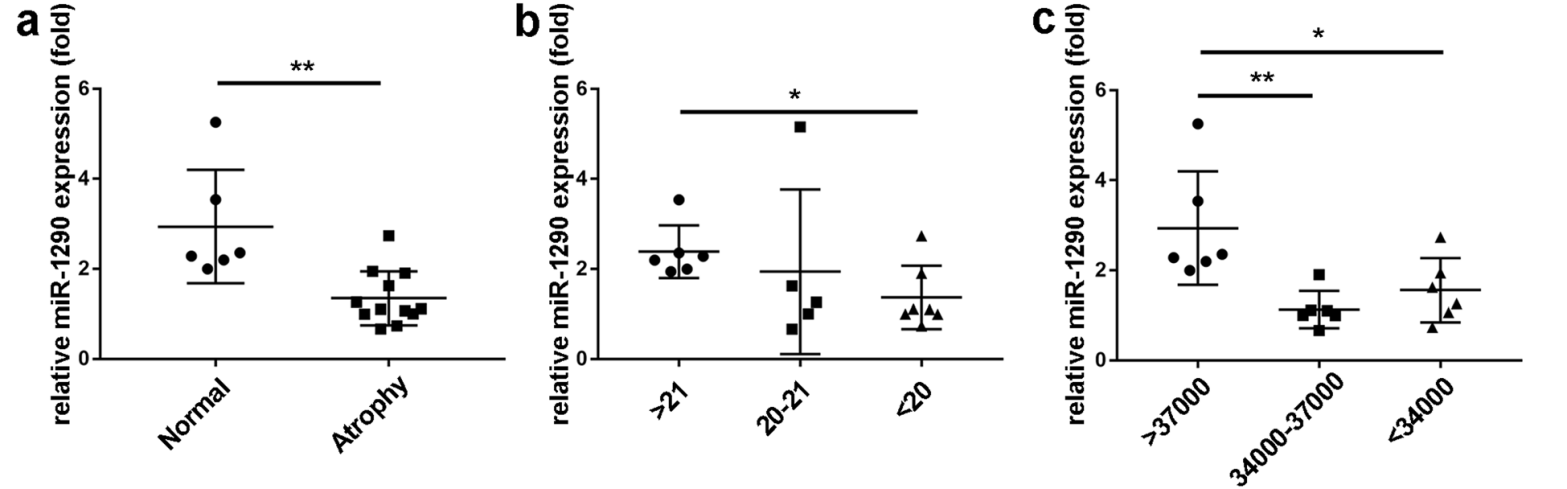
The correlation between miR-1290 and knee osteoarthritis patient with muscle atrophy. **a** RT-PCR result of miR-1290’s difference in serum. **b** and **c** The muscle strength and lean mass of different severity of muscle atrophy. The data was normalized to U6 and statistical difference among different group were considered significant at the levels of *P < 0.05, **P < 0.01, or ***P < 0.001

Through analyzing the serum expression of miR-1290 via RT-PCR, we concluded that the miR-1290 level of muscle atrophy patients was markedly lower compared with normal controls (Fig. 1a). To further assess the association between miR-1290 and the severity of muscle atrophy, we divided human subjects into three groups according to muscle strength (Q1: > 21 kg; Q2: 20–21 kg; Q3: < 20 kg) and lean mass (Q1: > 37,000; Q2: 34,000–37,000; Q3: < 34,000): Q1, Q2, and Q3 (Fig. 1b and c). The degree of miR-1290 reduction showed a negative correlation with the severity of atrophy.

### Effect of miR-1290 on C2C12 cell proliferation in vitro

**Fig. 2:**
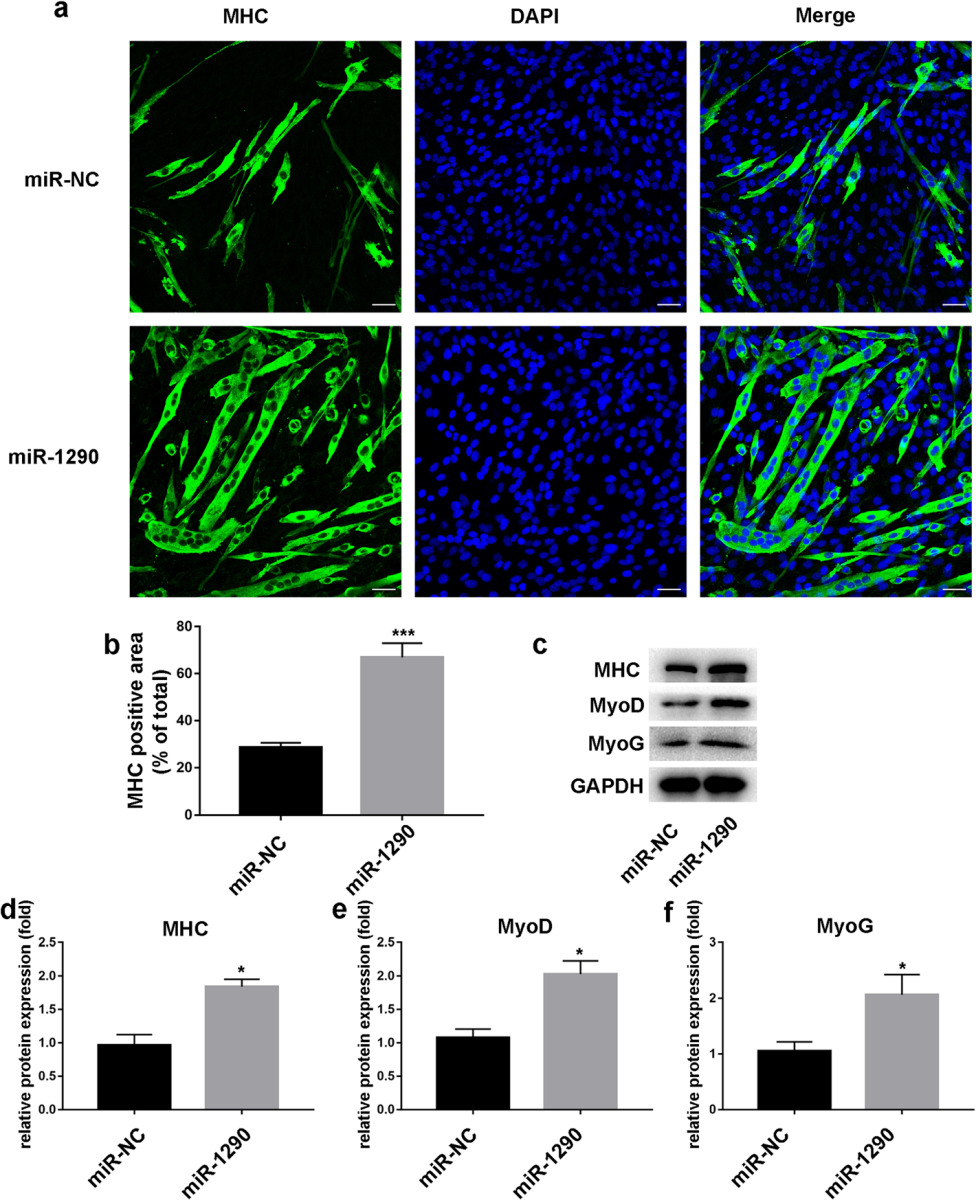
The effect of miR-1290 mimic transfection on C2C12 cells. **a** and **b** The MHC staining of C2C12 myoblast with miR-1290 or miR-NC and quantification of MHC area (scale bar, 50 μm). **c**–**f** The western blot analysis and quantification of MHC, MyoD, and MyoG after transfection of miRNAs. GAPDH was used as an internal control for western blot analysis. The statistical difference among miR-1290 transfection group and control group were considered significant at the levels of *P < 0.05, **P < 0.01, or ***P < 0.001

As shown in Fig. 2a, miR-1290 transfection led to an increase in the MHC-positive area in the MHC group compared with the control group. This result hinted that miR-1290 transfection increased differentiation in the C2C12 cells. In addition, the western blot results validated that miR-1290 transfection enhanced the protein levels of MHC, MyoD, and MyoG (Fig. 2c). These results revealed that differentiation in the miR-1290 transfection group was promoted compared with the group with normal C2C12 cells.

### Protective effect of miR-1290 in myotube atrophy in vitro

**Fig. 3:**
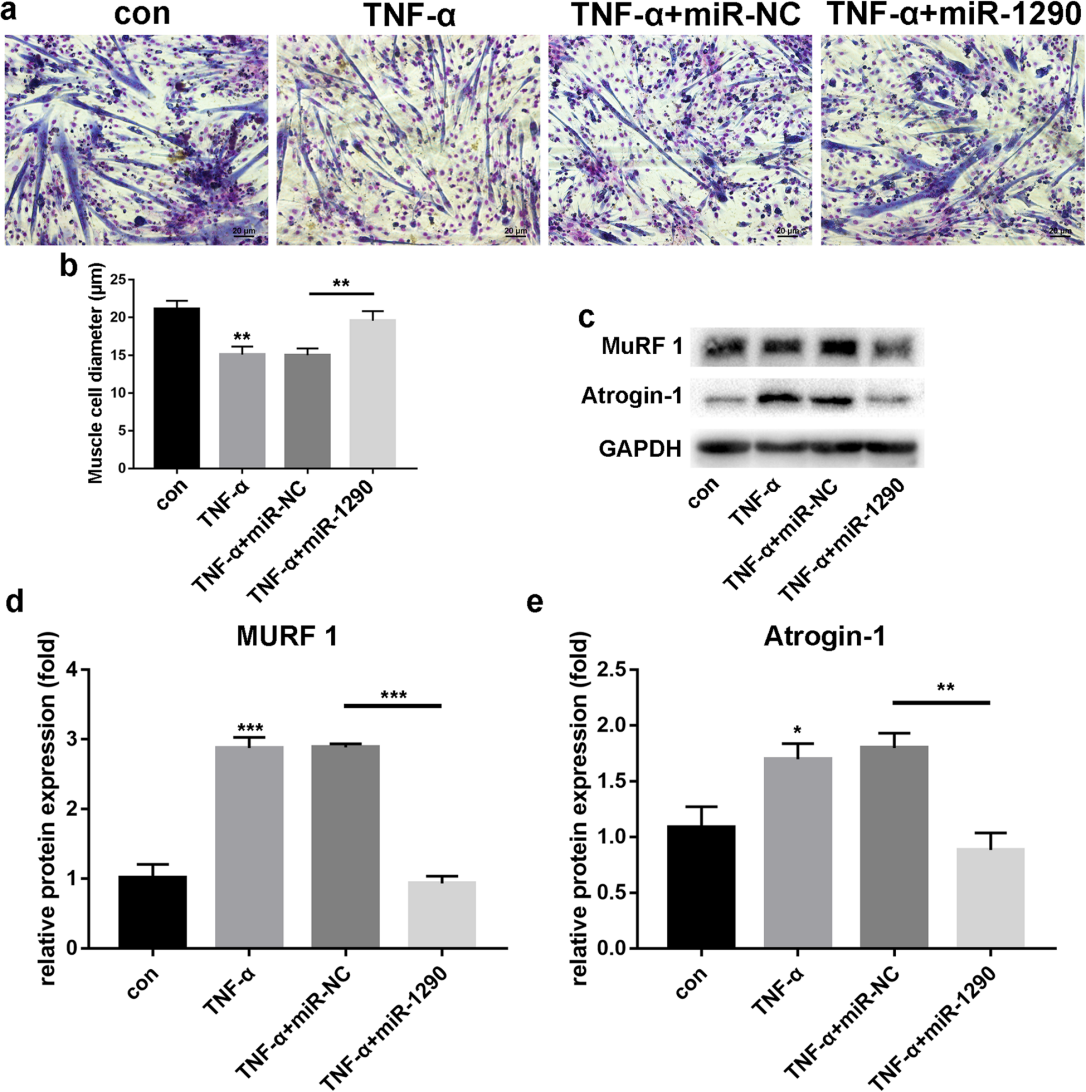
The effect of miR-1290 mimic transfection on C2C12 cells under TNF-α. **a** and **b** Giemsa staining and myotube diameters among all groups. **c**–**e** The western blot analysis and quantification of MuRF1 and atrogin-1 after overexpression of miR-1290 in TNF-α-induced atrophy. GAPDH was used as an internal control for western blot analysis. The statistical difference among miR-1290 transfection group and other groups were considered significant at the levels of *P < 0.05, **P < 0.01, or ***P < 0.001

To further explore the effect of miR1290 on muscle atrophy, we treated C2C12 myotubes with TNF-α for 24 h to establish a muscle atrophy model. Giemsa staining (Fig. 3a and b) was used to measure cell diameter. Compared with the normal control group (21.14 ± 1.07 μm), myotube diameters in the TNF-α stimulation group (15.11 ± 1.06 μm) were significantly decreased. However, myotube diameters increased in the miR-1290 transfection group (19.55 ± 1.26 μm). Furthermore, western blot analysis showed that the MuRF1 levels were increased in the TNF-α stimulation group, but decreased in the miR-1290 mimic group (Fig. 3c). Atrogin-1 showed similar trends (Fig. 3c). These results demonstrated the protective effect of miR-1290 in myotube atrophy in myoblast C2C12 cells.

### Protective effect of miR-1290 against myotube atrophy in vivo

**Fig. 4:**
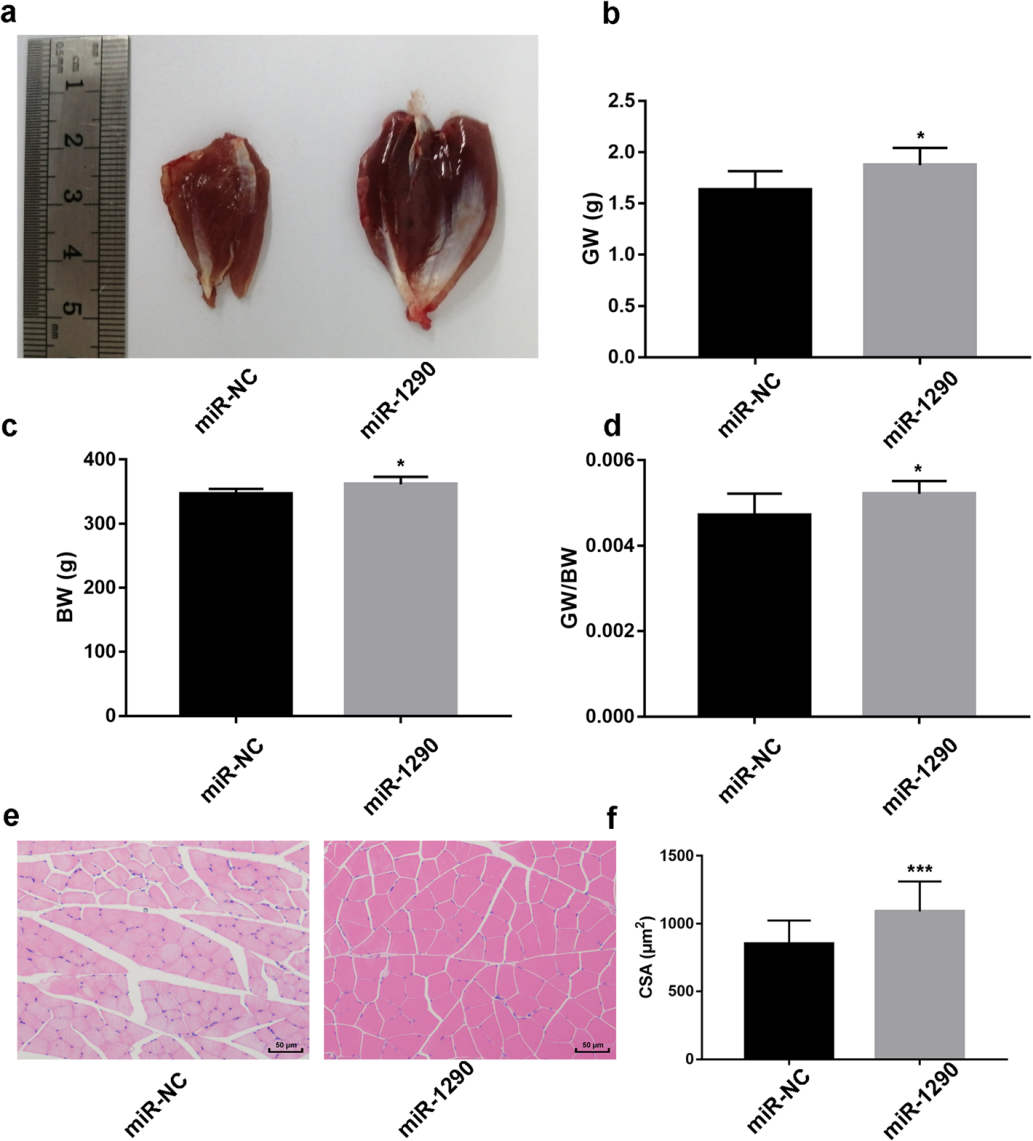
Effect of miR-1290 on muscle atrophy rat model. **a** The typical gastrocnemius muscle after injecting miR-NC and miR-1290 respectively in LPS-induced atrophy mice model. **b** The gastrocnemius muscle weight (GW) ratio after injecting miR-NC and miR-1290 respectively in LPS-induced atrophy mice model. **c** and **d** The body weight and the gastrocnemius muscle weight/body weight (GW/BW) ratio after injecting miR-NC and miR-1290 respectively in LPS-induced atrophy mice model. **e** HE staining after injecting miR-NC and miR-1290 respectively in LPS-induced atrophy mice model. **f** The muscle fiber cross-sectional area after injecting miR-NC and miR-1290 respectively in LPS-induced atrophy mice model. The statistical difference among miR-1290 mimic group and NC groups were considered significant at the levels of *P < 0.05, **P < 0.01, or ***P < 0.001

To validate the protective effect in vivo, LPS was employed to create a muscle atrophy model. The miRNA-1290 mimic injection attenuated the decrease in gastrocnemius muscle weight (GW), body weight (BW), and the gastrocnemius muscle weight/body weight (GW/BW) ratio (Fig. 4b–d). Moreover, HE staining revealed that LPS induced a marked decrease in the muscle fiber cross-sectional area, but miRNA-1290 mimic injection increased the muscle fiber cross-sectional area (Fig. 4e and f).

### MiR-1290 regulates C2C12 myogenesis and myotube atrophy by directly targeting FOXO3

**Fig. 5:**
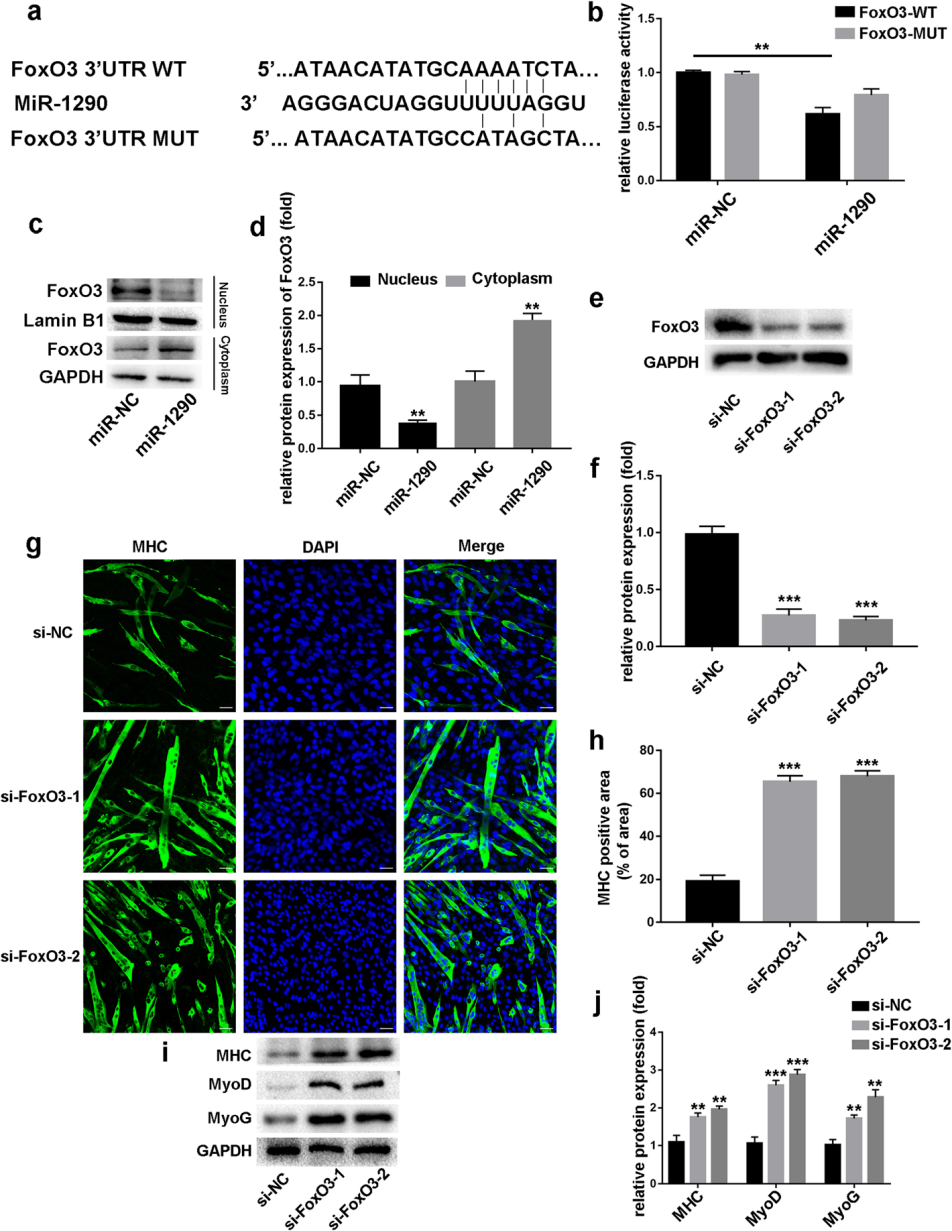
The intrinsic mechanism of miR-1290’s effect. **a** Bioinformatics analysis was performed to predict the miR-1290-binding seed sequence in the 3’UTR of FoxO3. **b** The luciferase result of miR-1290 and FOXO3. **c** and **d** The western blot analysis and quantification to determine FoxO3 levels in cytoplasm and nucleus in miR-1290-transfected C2C12 myoblast. **e** and **f** The knockdown efficiency of FoxO3-specific siRNA was confirmed by western blot. **g** and **h** MHC staining was performed after FoxO3 knockdown (scale bar, 50 μm). **i** and **j**. The expression of MyoD and MyoG were analyzed by western blot. GAPDH and Lamin B1 are cytoplasmic and nuclear protein loading controls, respectively. The statistical difference among miR-1290 transfection group and other groups were considered significant at the levels of * *P* < 0.05, **P < 0.01, or ***P < 0.001

To investigate the molecular mechanisms by which miR-1290 mediates C2C12 myogenic regulation and muscle atrophy, we hypothesized that miR-1290 may promote myogenic differentiation and inhibit myotube atrophy by directly targeting FOXO3. To explore whether miR-1290 directly targets FOXO3, we purchased wild-type (WT) and mutant (MUT) 3’UTR psi-CHECK2, which contained the miR-1290-binding site. Results showed that miR-1290 overexpression notably inhibited luciferase reporter activity from the vector containing the WT FOXO3 3’UTR, but mutated FOXO3 did not change significantly (Fig. 5b). Moreover, after transfecting miR-1290 mimics in C2C12 cells, we found gradually increasing FOXO3 expression levels in the cytoplasm and decreasing levels in the nucleus (Fig. 5c). To confirm whether FOXO3 affected myoblasts, siFOXO3 was transfected into C2C12 myoblasts. Western blot analysis showed that siFOXO3 inhibited FOXO3 expression successfully (Fig. 5e). The MHC-positive cell areas were markedly increased upon FOXO3 knockdown (Fig. 5g), and protein levels of MyoD and MyoG were also increased (Fig. 5i). Taken together, these results suggest that miR-1290 promotes myoblast differentiation by activating AKT/p70 pathway-mediated FOXO3 phosphorylation, which consequently prevents FOXO3 nuclear translocation.

### Role of protein kinase B (AKT)/P70/FOXO3 signaling pathway in miR-1290’s effect on myogenic differentiation

**Fig. 6:**
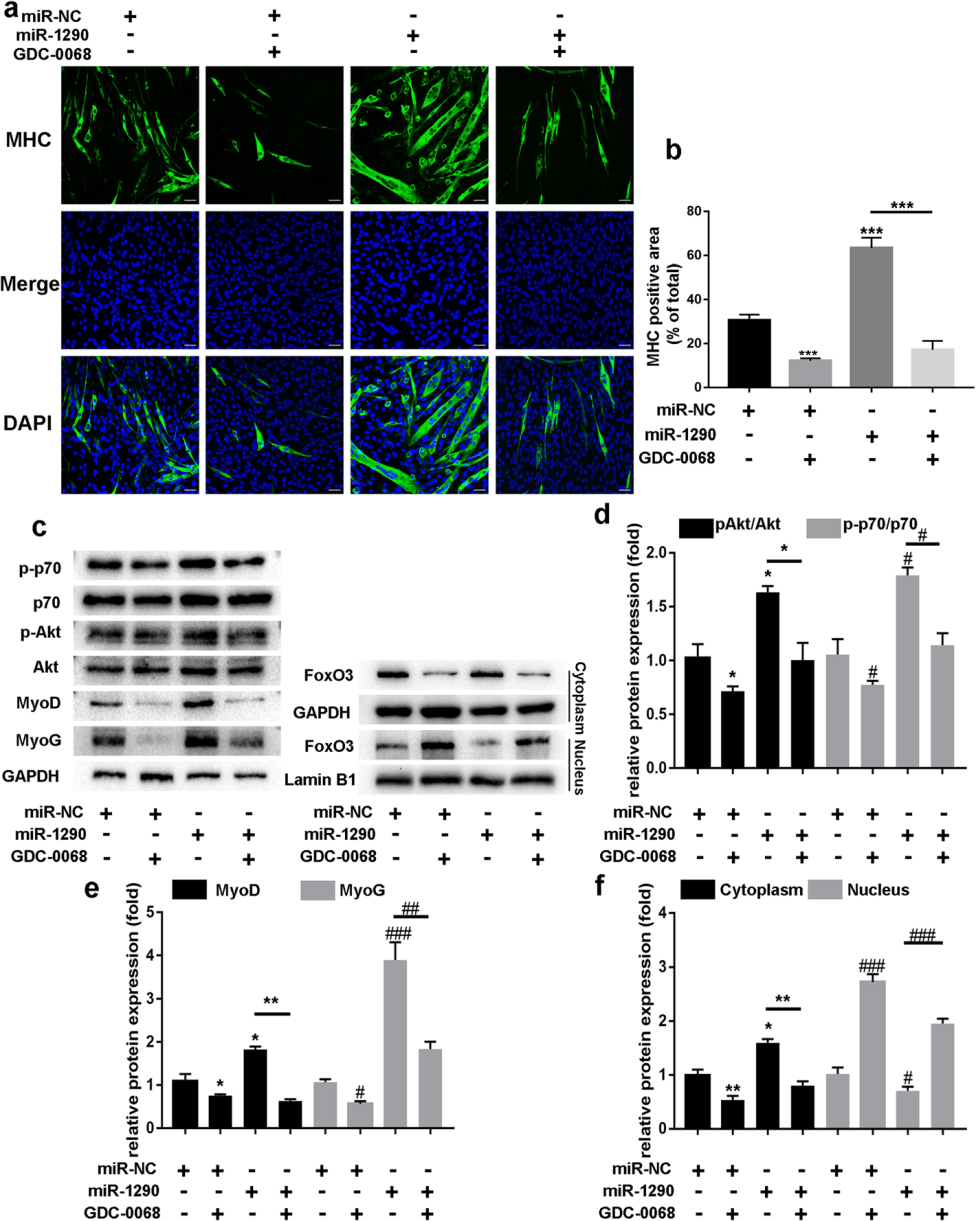
MiR-1290 activates AKT/P70/FoxO3 signaling pathways during myoblast differentiation. **a** MHC staining performed after treated miR-1290/miR-NC with or without GDC-0068 (scale bar, 50 μm). **b** MHC-positive areas/total areas were quantified using Harmony 4.1 software (*n* = 6). **c**–**f** The western blot analysis and quantification of phosphorylated and all forms of AKT and P70, MyoG, and MyoD, after transfecting miR-1290/miR-NC with or without GDC0068. GDC-0068 inhibited miR-1290-activated phosphorylation of AKT and P70 in C2C12 myoblasts. Western blot to analyze FoxO3 expression levels in cytoplasm and nucleus of C2C12 myoblasts. GAPDH and Lamin B1 are cytoplasmic and nuclear protein loading controls, respectively. The statistical difference among miR-1290 transfection group and inhibitor group were considered significant at the levels of *P < 0.05, **P < 0.01, or ***P < 0.001

It is known that activation of AKT induces FOXO3 phosphorylation, which maintains the transcription factor in an inactive state in the cytoplasm. As shown in Fig. 6c, the phosphorylation protein levels of AKT and P70 were increased in the miR-1290 transfection group, which confirmed that miR-1290 may promote myoblast differentiation via AKT/P70/FOXO3 signaling pathway modulation. Next, we added AKT inhibitor (GDC-0068) to C2C12 myotubes. Western blot analysis showed decreased expression of MyoD and MyoG, as well as decreased MHC measured by IF (Fig. 6a and c). However, miR-1290 transfection did not increase the expression of MHC, MyoD, and MyoG after GDC-0068 treatment. Furthermore, after adding GDC-0068, miR-1290 did not increase the phosphorylation of AKT and P70 (Fig. 6c). In C2C12 myoblasts, we observed increased FOXO3 protein in the cytoplasm and a decreased level in the nucleus after miR-1290 transfection. However, combination treatment of miR-1290 and GDC-0086 did not increase FOXO3 in the cytoplasm or decrease FOXO3 in the nucleus of C2C12 myoblasts (Fig. 6c). Taken together, we conclude that miR-1290 promotes myogenesis by activating AKT/P70 signaling-mediated FOXO3 nuclear translocation.

### Role of protein kinase B (AKT)/P70/FOXO3 signaling pathway in miR-1290’s effect on myotube atrophy

**Fig. 7:**
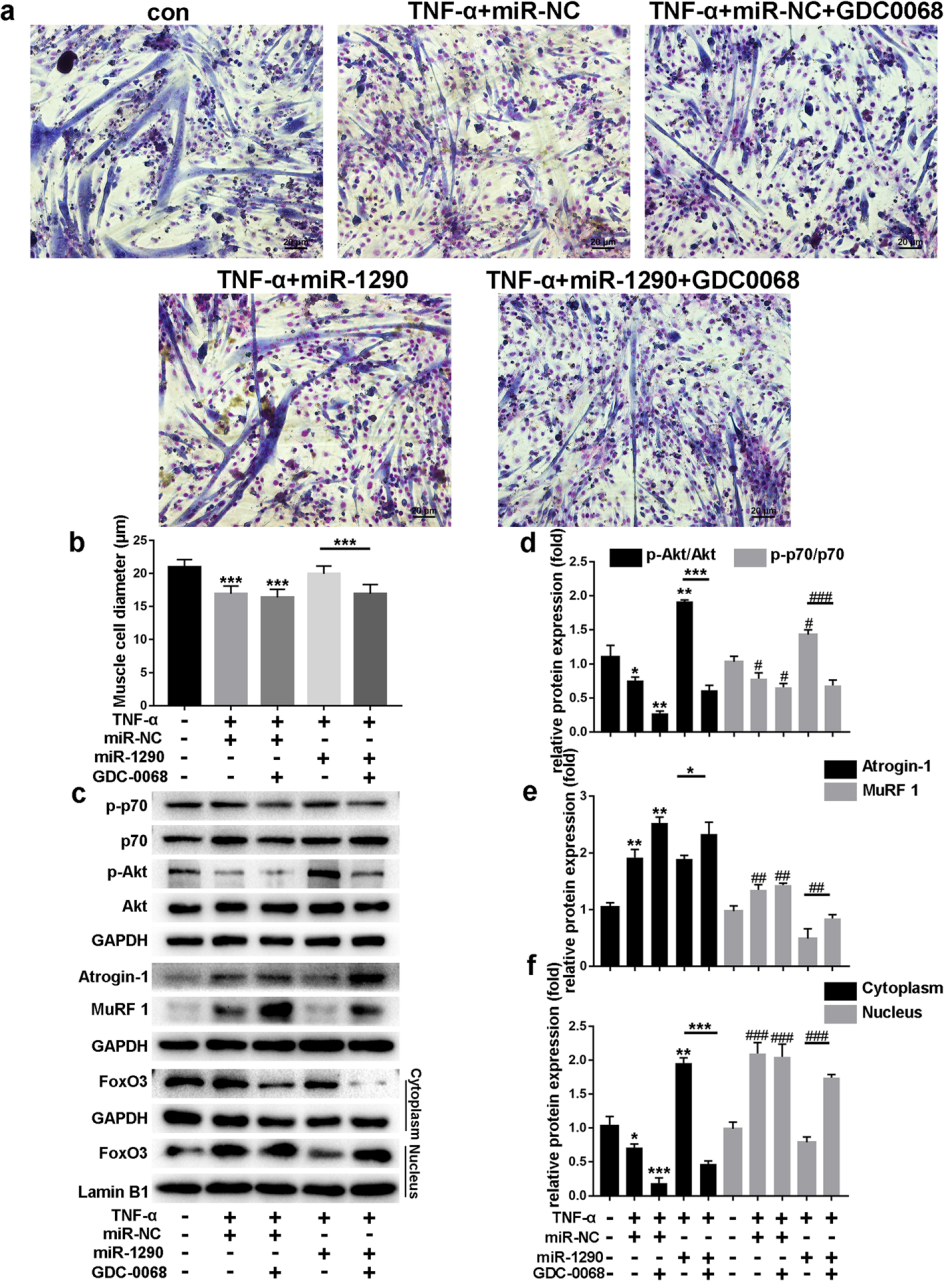
Role of Protein kinase B (AKT)/P70/FOXO3 signaling pathway in effect of miR-1290 on myotube atrophy. **a** Giemsa staining was performed to calculate myotube diameters for TNF-α + miR-NC or TNF-α + miR-NC + GDC0068, TNF-α + miR-1290 or TNF-α + miR-1290 + GDC0068 treatments. **b** Cell diameters of five groups were measured. **c**–**f** Western blot analysis and quantification of phosphorylated and all forms of AKT and P70 of C1C12 myotubes for TNF-α + miR-NC or TNF-α + miR-NC + GDC0068, TNF-α + miR-1290 or TNF-α + miR-1290 + GDC0068 treatments. Western blot was performed to analyze the expression of MuRF1 and atrogin-1 in TNF-α + miR-NC or TNF-α + miR-NC + GDC0068, TNF-α + miR-1290 or TNF-α + miR-1290 + GDC0068 groups. After treatment with miR-1290/miR-NC or with GDC-0068, FoxO3 expression levels in cytoplasm and nucleus of C2C12 myotubes were examined by western blot. GAPDH and Lamin B1 are cytoplasmic and nuclear protein loading controls, respectively. The statistical difference among miR-1290 transfection group and inhibitor group were considered significant at the levels of *P < 0.05, **P < 0.01, or ***P < 0.001

To explore the effect of miR-1290 on muscle atrophy, we measured myotube diameters after treating TNF-α-induced C2C12 myotubes with miR-1290 and/or GDC-0068 (Fig. 7a). As shown in Fig. 7c, miR-1290 significantly increased the phosphorylation of both AKT and P70 and decreased the expression of MuRF1 and atrogin-1 even after TNF-α treatment, while this ability disappeared after GDC-0086 treatment. Moreover, miR-1290 transfection did not activate FOXO3 phosphorylation or promote translocation of FOXO3 from the nucleus to the cytoplasm after inhibiting the AKT pathway (Fig. 7c).

## Discussion

This study was designed to investigate the role of miR-1290 in C2C12 myoblast cells. In this study, miR-1290 expression accelerated the differentiation of C2C12 cells. The AKT/P70/ FoxO3 signaling pathway played crucial roles in the myoblast differentiation Importantly, the promoted differentiation and protective effect against atrophy were validated using a rat atrophy model.

The proliferation and differentiation of muscle cells are important biological processes in skeletal myogenesis, and foremost factors against muscle atrophy [19]. When proliferation and differentiation are insufficient, muscle atrophy occurs when the rate of protein degradation in body tissue exceeds the rate of protein synthesis. The differentiation stage of myoblast cells is a complex process regulated by specific transcription factors [20]. First, proteins related to the cell cycle, such as cyclin D1, cyclin E, and CDK4, can promote cell proliferation in normal and cancer cells [17]. Meanwhile, transcription factors such as MYF5 (Myogenic factor 5), MYF6 (Myogenic factor 6), MyoD (Myogenic differentiation antigen), MyoG (Myogenin), and MRF4 (Myogenic regulatory factor 4), also called the myogenic regulatory factor family, can regulate differentiation into myotubes [21, 22]. The pro-inflammatory cytokine TNF-α elicits muscle proteolysis and blocks myogenesis by destabilizing MyoD [23]. In this study, TNF-α downregulated the myoD genes and protein levels. However, the MyoD and MyoG genes, and protein levels were enhanced after miR-1290 transfection. Therefore, miR-1290 promoted the differentiation process.

Several proteins and signaling pathways participate in the regulation of the myoblast differentiation process, such as AKT, p70, AMP-activated protein kinase (AMPK), FoxO3, IGF-1, and TGF-β [24, 25]. AKT and its downstream p70 can receive upstream stimuli, such as IGF-1, during myoblast differentiation and muscle mass maintenance and activation [26]. A previous report indicated that phosphorylation of AKT/mTOR blocked FoxO3 translocation to the nucleus, and therefore, muscle atrophy was inhibited [27]. The mTOR phosphorylation also elicited the downstream targets such as YY1. Consequently, the AKT pathway regulated energy homeostasis processes in myoblasts, including intracellular ATP synthesis, citrate synthase activity, glycolysis, mitochondrial DNA copy maintenance, gene transcription, and protein translation, which are linked to energy metabolism [28]. In this study, an AKT inhibitor, GDC-0068, decreased the MHC-positive ratio compared with the miR-1290 transfection group, and downregulated the MyoD and MyoG genes and proteins levels, thus validating the crucial role of AKT in miR-1290’s regulatory effect on C2C12 cells. The transcription factor FOXO3 is one member of the human FoxO family [29]. FOXO3 is widely expressed in skeletal muscle tissue and participates in numerous cellular responses such as autophagy, apoptosis, stem cell homeostasis, and ROS diminishing [30]. FOXO3 activation in the nucleus accelerates the upregulation of Mafx and Murf1 mRNA [31], as well as MAFbx and HDAC6 proteins [32]. In this study, FOXO3 was found to be the target of miR-1290.

MiR-1290 is a miRNA that often contributes to cancer development. MiR-1290 accelerated cell proliferation and invasion in the pathological processes of gastric cancer [33], esophageal squamous cell carcinoma [34], lung cancer [13], pancreatic carcinoma [35], and colorectal cancer [36]. Therefore, miR-1290 was recognized as a cancer biomarker [37], and thus a potential therapeutic target of anti-cancer compounds [38]. Additionally, miR-1290 expression enhanced the production of pulmonary fibrosis markers. Most importantly, miR-1290 accelerated the differentiation of hepatocyte-like cells from mesenchymal stem cells [39]. This report hinted at the possibility of miR-1290’s effect on myoblast cells. In this study, we found that the miR-1290 mimic successfully promoted myoblast cell differentiation and exerted a protective effect against muscle atrophy in vivo. Previous reports revealed that targets of miRNA contained the BY targeting nuclear factor I/X [34], IkB kinase complex (IKK1) [40], suppressor of cytokine signaling 4 (SOCS4) [41], interferon regulatory factor 2 [42], LHX6 [43], and inositol polyphosphate 4-phosphatase B (INPP4B) [44]. Our study is the first to reveal that FOXO3, a critical skeletal muscle myogenesis regulation protein, is the target of miR-1290.

## Conclusion

In summary, the present study revealed the role of miR-1290 in myoblast cell differentiation and muscle atrophy. The miR-1290 mimics accelerated C2C12 cell differentiation. The AKT/P70/ FoxO3 signaling pathway plays crucial roles in the effect of miR-1290 in myogenesis. The promoted differentiation and protective effects on atrophy were validated using a rat atrophy model.

## Data Availability

The datasets used and/or analyzed during the current study are available from the corresponding author on reasonable request.

## Abbreviations

AIDS: Acquired immunodeficiency syndrome
miRNAs: MicroRNAs
FOXO3: Forkhead Box O3
AKT: Protein kinase B
PBS: Phosphate-buffered saline
DMEM: Dulbecc o’s Modified Eagle’s Medium
MHC: Myosin heavy chain
MyoG: Myogenin
MuRF1: Muscle-specific RING Finger 1
IF: Immunofluorescence
qPCR: Real-time polymerase chain reaction
SD: Sprague Dawley
SPF: Specific pathogen-free
NC: Negative control
BW: Body weight
GW: Gastrocnemius weight
TNF-α: Tumor necrosis factor alpha
HE: Hematoxylin–eosin
SD: Standard deviation
WT: Wild-type
MYF5: Myogenic factor 5
MYF6: Myogenic factor 6
MyoD: Myogenic differentiation antigen
MRF4: Myogenic regulatory factor 4
AMPK: AMP-activated protein kinase
IGF-1: Insulin-like growth factor 1
TGF-β: Transforming growth factor-β
mTOR: Mammalian target of rapamycin
ROS: Reactive oxygen species
IKK1: IkB kinase complex
SOCS4: Suppressor of cytokine signaling 4
INPP4B: Inositol polyphosphate 4-phosphatase B
